# The Effects of Zinc and Selenium Co-Supplementation on Resting Metabolic Rate, Thyroid Function, Physical Fitness, and Functional Capacity in Overweight and Obese People under a Hypocaloric Diet: A Randomized, Double-Blind, and Placebo-Controlled Trial

**DOI:** 10.3390/nu15143133

**Published:** 2023-07-13

**Authors:** Antonis Zavros, Eleni Andreou, George Aphamis, Gregory C. Bogdanis, Giorgos K. Sakkas, Zoe Roupa, Christoforos D. Giannaki

**Affiliations:** 1 Department of Life Sciences, University of Nicosia, Nicosia 2417, Cyprus; zavrosantonis@gmail.com (A.Z.); andreou.el@unic.ac.cy (E.A.); aphamis.g@unic.ac.cy (G.A.); roupa.z@unic.ac.cy (Z.R.); 2 Research Centre for Exercise and Nutrition (RECEN), Nicosia 2417, Cyprus; 3 School of Physical Education and Sport Science, National and Kapodistrian University of Athens, 10679 Athens, Greece; gbogdanis@phed.uoa.gr; 4 Department of Physical Education and Sport Science, University of Thessaly, 42100 Trikala, Greece; gsakkas@uth.gr

**Keywords:** free triiodothyronine, thyroid-stimulating hormone, free thyroxine, body fat, fat-free mass, exercise performance, muscle mass, antioxidants, body mass index

## Abstract

Evidence of the effectiveness of zinc (Zn) and selenium (Se) on resting metabolic rate (RMR) and physical function parameters in people with overweight and obesity is scarce, while the effects of zinc and selenium on thyroid function and body composition are still a topic of debate and controversy. The aim of this randomized, double-blind, and placebo-controlled trial was to examine the effects of a hypocaloric diet and Se–Zn co-supplementation on RMR, thyroid function, body composition, physical fitness, and functional capacity in overweight or obese individuals. Twenty-eight overweight–obese participants (mean BMI: 29.4 ± 4.7) were randomly allocated (1:1) to the supplementation group (*n* = 14, 31.1 ± 5.5 yrs, 9 females) and the placebo group (*n* = 14, 32.1 ± 4.8 yrs, 6 females). The participants received Zn (25 mg of zinc gluconate/day) and Se (200 mcg of L-selenomethionine/day) or placebo tablets containing starch for eight weeks. The participants of both groups followed a hypocaloric diet during the intervention. RMR, thyroid function, body composition, cardiorespiratory fitness (VO_2_max), and functional capacity (sit-to-stand tests, timed up-and-go test, and handgrip strength) were assessed before and after the intervention. A significant interaction was found between supplementation and time on RMR (*p* = 0.045), with the intervention group’s RMR increasing from 1923 ± 440 to 2364 ± 410 kcal/day. On the other hand, no interaction between supplementation and time on the thyroid function was found *(p* > 0.05). Regarding the effects of Zn/Se co-administration on Se levels, a significant interaction between supplementation and time on Se levels was detected (*p* = 0.004). Specifically, the intervention group’s Se serum levels were increased from 83.04 ± 13.59 to 119.40 ± 23.93 μg/L. However, Zn serum levels did not change over time (90.61 ± 23.23 to 89.58 ± 10.61 umol/L). Even though all body composition outcomes improved in the intervention group more than placebo at the second measurement, no supplement × time interaction was detected on body composition (*p* > 0.05). Cardiorespiratory fitness did not change over the intervention. Yet, a main effect of time was found for some functional capacity tests, with both groups improving similarly over the eight-week intervention period (*p* < 0.05). In contrast, a supplement x group interaction was found in the performance of the timed up-and-go test (TUG) (*p* = 0.010), with the supplementation group improving more. In conclusion, an eight-week intervention with Zn/Se co-supplementation combined with a hypocaloric diet increased the RMR, TUG performance, and Se levels in overweight and obese people. However, thyroid function, Zn levels, body composition, and the remaining outcomes of exercise performance remained unchanged.

## 1. Introduction

According to the World Health Organization, the number of obese people has almost tripled since 1975 [1]. More specifically, in 2016 the number of overweight and obese adults aged 18 years and over was 1.9 billion and 650 million, respectively. Obesity is associated with more than 50 pathological conditions [2]. In particular, obesity can have an overall clinical impact leading to poor health, disability, and premature death, and also a psychological impact reducing the quality of life and increasing the risk for social withdrawal and isolation [2].

The causes of obesity are complex and can be classified as primary and secondary, with an interaction between biological, behavioral, and psychosocial factors taking place [3]. Even though it is debatable [4], in 2013 obesity was categorized by the American Medical Association as a disease. That is because it is not only characterized by excessive fat accumulation but also by pathological and functional disturbances of the adipose tissue, known as adiposopathy [5]. These disturbances include adipocyte hypertrophy, visceral adiposity, and/or ectopic fat deposition and could be the causes of metabolic disease development [6]. Adipocytes and macrophages are cells involved in the production of molecules called adipocytokines [7]. It is believed that an increased adipose tissue, as a result of adipocyte hypertrophy, enriches the production of adipocytokines, which can lead to pathophysiological processes related to inflammation [7,8].

Growing evidence confirms that obesity increases the risk for nutritional deficiencies due to poor diet quality and a disruption of the bioavailability and metabolism of numerous nutrients despite the high caloric intake that characterizes the specific population [9,10,11,12]. Notably, in people with obesity undergoing a hypocaloric diet, a formula providing 100% of the Daily Recommended Intake could not prevent the nutritional deficiencies in some micronutrients [13]. That is important because micronutrient deficiencies negatively affect the body’s health and are closely related to the development of multiple diseases [14,15]. As a result, nutritional supplements containing minerals and vitamins combined with lifestyle changes have been proposed in some studies for obesity therapy and populations with a high risk for deficiencies [9,14,15].

A systematic review of the literature indicated that people with obesity have lower levels of some antioxidants, including zinc (Zn) and selenium (Se) [16]. Zn is a trace element involved in the metabolism of obesity-related hormones like insulin, leptin, and thyroid hormones and has a role in most metabolic pathways [16,17,18,19]. On the other hand, Se is considered an essential micronutrient for humans and plays biological roles by forming 25 selenoproteins synthesized by the Se metabolic system [20,21]. With its effects on insulin signaling, Se can modulate fat and lipid metabolism [22,23].

Numerous systematic reviews and meta-analyses have highlighted that Se and Zn supplementation may positively affect many aspects of health in people with or without diseases and pathological conditions [24,25,26,27,28]. Regarding the effects of Zn and Se supplementation on body composition, positive results have been found in some animal studies [29,30,31,32]. However, according to a systematic review, randomized controlled trials about the effects of the two elements on body composition in people with overweight or obesity are conflicting and have several limitations [33]. Therefore, more randomized controlled trials are needed so that the effects of Zn and Se on individuals with overweight or obesity can be revealed [33].

Even though a diet plan can induce weight loss by creating a negative energy balance, maintaining weight loss can sometimes be challenging in the long term [34]. A possible explanation for this phenomenon might be the decrease in energy expenditure [35,36] and thyroid hormone levels [37,38,39] sometimes observed with weight loss. Moreover, previous studies demonstrated that when adjusted for body weight, the resting metabolic rate (RMR) of obese children is lower than that of normal-weight children [40] and that people with higher BMI have lower-than-predicted RMR [41]. Since RMR contributes to approximately 70% of Total Daily Energy Expenditure, decreases in RMR, as mentioned above, could have a negative impact on weight loss and weight maintenance.

Two studies examining the effects of Zn on human RMR or Basal Metabolic Rate (BMR) showed some positive results [42,43]. The BMR of six men decreased significantly by the end of a low-Zn diet and tended to increase by the end of a diet with adequate Zn [42]. The Zn-adequate diet also affected the thyroid function of the participants by increasing their serum thyroid-stimulating hormone (TSH), thyroxine (T4), and free T4 (FT4) [42]. The second study was a case–control by Maxwell and Volpe [43]. After four months of Zn supplementation in two female Zn-deficient participants, their RMR and triiodothyronine (T3) levels increased [43].

Hawkes and Keim [44] reported the effects of high- and low-Se diets on human RMR. In their study, a sample of healthy men was divided into two groups and fed foods high in Se or low in Se for four months. Even though the RMR at the end of the study was not different between the groups, serum T3 decreased in the high-Se group, increased in the low-Se group, and significantly differed between groups from day 45 onwards. A significant increase in TSH occurred only in the high-selenium group, probably indicating a hypothyroid response [44]. However, the negative impact of the high-Se diet on thyroid function could be attributed to dietary components other than Se [45]. That is because, in a later study on healthy men, the participants’ thyroid hormone levels remained unchanged, with a tendency toward higher T3 levels after twelve months of Se yeast supplementation [45]. To our knowledge, no randomized controlled trial has ever examined the effects of Zn and/or Se supplementation on RMR of overweight or obese people. In addition, randomized controlled trials investigating the effects of Zn and/or Se on thyroid function in euthyroid individuals with overweight or obesity are scarce [33].

In several studies, the low RMR of individuals with obesity was associated with lower cardiorespiratory fitness levels, probably due to limited physical activity levels [40,41]. It appears that many obese people exhibit reduced physical performance and have exercise-related constraints, probably as a result of pathophysiological changes which occur from the increased body mass [46]. The reduced level of daily physical activities due to the low fitness level is associated with several risk factors for cardiovascular disease [47,48]. Though there is not enough evidence to support a causal effect, the micronutrient deficiencies observed in people with obesity might be partly responsible for their reduced exercise performance since they are related to a decline in muscle mass, strength, and physical performance [49,50].

A recent review found that a poor Zn status in healthy individuals, as a result of a dietary consumption below the recommended daily allowances (RDA), can negatively affect their physical performance [51]. However, studies included in that review did not provide any data on Zn intake at levels higher than the RDA [51]. As such, it was concluded that there was not enough evidence in support of any beneficial effects of Zn supplementation beyond RDA levels on physical performance in humans. On the other hand, there is emerging research regarding the mechanisms via which Se affects skeletal muscle health and mitochondrial function, emphasizing the role of Se beyond an antioxidant [52]. Furthermore, Se is associated with muscle fatigue, with low Se levels explaining part of the decrease in performance during exercise [53].

The aim of this randomized control trial was to examine the effects of Zn and Se co-supplementation on RMR, thyroid function, exercise performance, and body composition in people with overweight or obesity undergoing a hypocaloric diet.

## 2. Materials and Methods

### 2.1. Study Design

**Figure 1:**
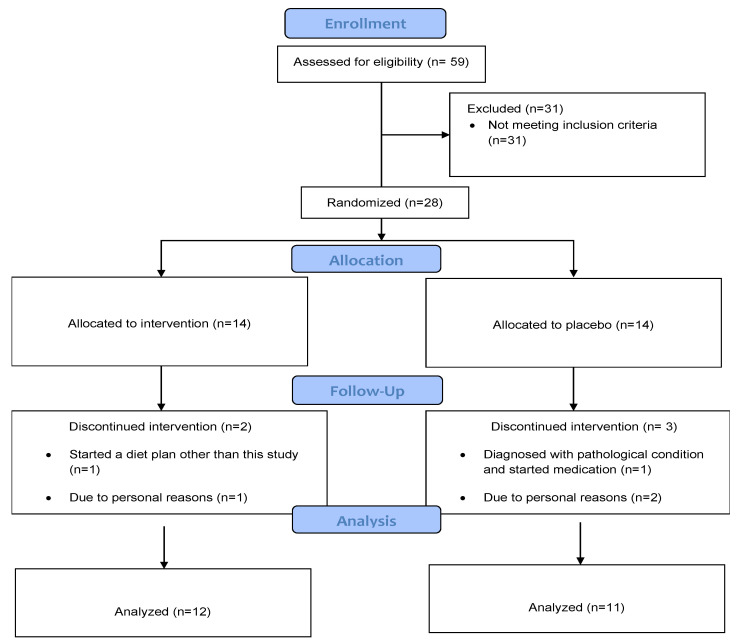
Consort 2010 Flow Diagram.

This was a randomized, double-blind, and placebo-controlled trial involving adult men and women with overweight or obesity (BMI ≥ 25). After the initial screening, twenty-eight participants (mean BMI: 29.4 ± 4.7) were randomly allocated (1:1) to the supplementation group (*n* = 14, 31.1 ± 5.5 yrs, 9 females, 5 males) and the placebo group (*n* = 14, 32.1 ± 4.8 yrs, 6 females, 8 males). During the intervention period, two participants from the intervention group and three participants from the placebo group dropped out of the study. The reasons are shown in the Consort Flow Diagram (Figure 1). The study was approved by the Cyprus National Ethics Committee (ΕΕΒΚ/ΕΠ/2020/18) and was in accordance with the Declaration of Helsinki. Inclusion criteria for the study were: (a) overweight or obese men and women (ΒΜΙ ≥ 25); (b) age 18–40 years; (c) no diet intervention during at least the last three months; and (d) no use of nutritional supplements/medications before (≥3 months) and during the study. Exclusion criteria were: (a) history of thyroid disease, positivity for thyroid autoantibodies, or treatment with medications potentially interfering with thyroid function; (b) diagnosis of cardiovascular, metabolic, pulmonary, renal, musculoskeletal, or mental disorders; and (c) pregnancy or lactation. In addition, a medical doctor, member of the research group, examined the participants to ensure that no one would be exposed to high risk.

### 2.2. Intervention

Participants in the supplementation group received 25 mg/day of Zn gluconate and 200 mcg/day of Se L-selenomethionine and the controlled group received placebo supplements of identical color and shape, containing starch (Figure 1). The supplements were consumed immediately after lunch. Similar Se and Zn dosages were used in previously published research with overweight or obese people [54,55,56,57]. Moreover, these dosages can be considered safe, as they are lower than the tolerable upper limit intake levels for adults (40 mg/day for Zn and 400 mcg/day for Se). After a nutritional assessment by a registered dietitian (member of the research group), the participants of both groups received a balanced, restricted-calorie diet with 300 kcal lower than the estimated energy requirement based on each participant’s RMR and physical activity levels. Adherence to the diet was assessed every month by a registered dietitian. In addition, participants were contacted via telephone calls to ensure their compliance and were asked to maintain their usual physical activity level.

### 2.3. Blinding and Randomization

A table of random numbers was used so each participant could have the same chance of receiving either the Zn–Se supplement or placebo tablets. This way, the investigators could not predict which treatment was next. In addition, as the placebo tablets were identical to the supplements, neither the investigator doing the assessments nor the study participants could identify to which study group the participant belonged. 

### 2.4. Anthropometry and Body Composition 

Weight was measured using a calibrated scale (Seca, Hamburg, Germany) with minimum clothes and without shoes. Height was measured without shoes by the use of a wall-mounted stadiometer with a precision of 0.5 cm. BMI was estimated using the formula weight (kg)/height^2^ (m^2^). Body mass, body fat, and lean body mass were measured using multi-frequency bioimpedance technology (Tanita, MC980Uplus, Tanita Corp., Tokyo, Japan).

### 2.5. Participants’ Adherence 

The participants’ adherence to the supplements was estimated by the number of remaining tablets. Participants’ data were analyzed if the compliance was ≥90%.

### 2.6. Thyroid Function Assessment

Thyroid hormones were assessed biochemically by measuring serum TSH, FT3, and FT4 concentration [58,59,60]. Se and Zn serum concentrations were also evaluated. Blood sampling was carried out by specialized nursing staff accredited to perform phlebotomy. Blood analyses were carried out in an accredited laboratory with appropriate analytical assays.

### 2.7. Resting Metabolic Rate

RMR was estimated using indirect calorimetry (Quark CPET, Cosmed, Rome, Italy). The tests were conducted early in the morning, with participants lying quietly on a reclined bed for 15 min in a dark, quiet room. Then, the respiratory face mask was placed, measuring RMR for the remaining 15 min. All participants abstained from strenuous exercise for at least 48 h before measurement and from eating for 12 h prior to measurement [61]. The metabolic cart was switched on 30 min before the measurement and was calibrated before each measurement.

### 2.8. Functional Capacity and Physical Fitness Assessment

Functional capacity was assessed by a battery of functional tests, including two versions of the “Sit-to-Stand Tests” (STS-5 and STS-60) and the “Timed Up-and-Go Test” [62,63]. The Timed Up-and-Go test evaluates agility, dynamic balance, and functional mobility and requires patients to stand up out of a chair, walk 3 m, turn around, walk back to the chair, and sit down. The STS-5 requires the patients to perform five sit-to-stand cycles as fast as possible, measured in seconds, and can be used as an indicator of the patient’s lower extremities strength. The STS-60 is a similar test, requiring the patient to stand up and sit down on a chair as many times as possible in 60 s. The score is the total number of sit-to-stand cycles within 60 s (the number achieved in 30 s was also recorded), and it is an index of muscular endurance. Handgrip strength was assessed using a digital hand-held dynamometer (T.K.K. 5401 Grip—D; Takey, Japan). First, both arms were relaxed, in the neutral position by the side of the body. Then, participants were asked to hold and squeeze the dynamometer for 3 s. No other body movement was allowed. The measurement was performed by both the right and left arms.

Maximal oxygen uptake (VO_2_max) was determined from an incremental cycling test on a cycle ergometer (LC6, Monark, Sweden) using a breath-by-breath gas analysis (Quark CPET, Cosmed, Rome, Italy). The initial workload of the incremental was set at 50 W and was increased by 25 W every minute until the participants reached volitional exhaustion or could not maintain a cadence of 60 rpm and the respiratory exchange ratio was above 1.1. Heart rate was continuously monitored with a Polar heart rate monitor (Polar^®^ H7, Polar Electro Oy^®^, Kempele, Finland). The tests were performed by the exercise physiologists, members of the research team.

### 2.9. Statistical Analysis

The Kolmogorov–Smirnov test was used to assess normality of distribution. Repeated measures ANOVA (group × time) were used to analyze the results. Tukey post hoc tests were used to determine pairwise differences wherever repeated measures ANOVA showed statistical significance *p* ≤ 0.050. Statistical significance was set at *p* ≤ 0.050, and all results were reported as mean ± standard deviation. The statistical analysis was performed using the SPSS program (IBM SPSS Statistics version 21; SPSS Inc., Chicago, IL, USA).

## 3. Results

No side effects of the supplementation were reported during the 8-week intervention while all participants showed a compliance of >90%. No differences were found in any examined variable between the two groups at baseline.

### 3.1. Effects on Anthropometry and Body Composition

The results for anthropometry and body composition parameters are presented in Table 1 No significant interaction effect between group and time was found regarding body weight (F_1, 20_ = 0.08, *p* > 0.05), and no main effect of group (F_1, 20_ = 0.29, *p* > 0.05) or assessment times (F_1, 20_ = 0.09, *p* > 0.05) was observed. Body fat was not affected following the intervention period. In particular, no significant interaction effect between group and time was found (F_1, 15_ = 2.31, *p* > 0.05), and no main effect between groups was observed (F _1, 15_ = 0.19 *p* > 0.05). However, a main effect between assessment times was detected (F_1, 15_ = 7.18, *p* = 0.014), with participants having 0.99% less body fat in the second assessment as compared to the beginning of the study. Regarding fat-free mass, no significant interaction effect between group and time was found, F_1, 20_ = 1.90, *p* > 0.05. Furthermore, no main effect between groups (F_1, 20_ = 0.05, *p* > 0.05) or assessment times (F_1, 20_ = 0.48, *p* > 0.05) was observed. The results for muscle mass showed no significant interaction effect between group and time, F_1, 20_ = 1.87, *p* > 0.05. Moreover, no main effect of groups (F_1, 20 =_ 0.05, *p* > 0.05) or assessment times (F_1, 20_ = 0.47, *p* > 0.05) was found.

### 3.2. Effects on Resting Metabolic Rate

**Figure 2:**
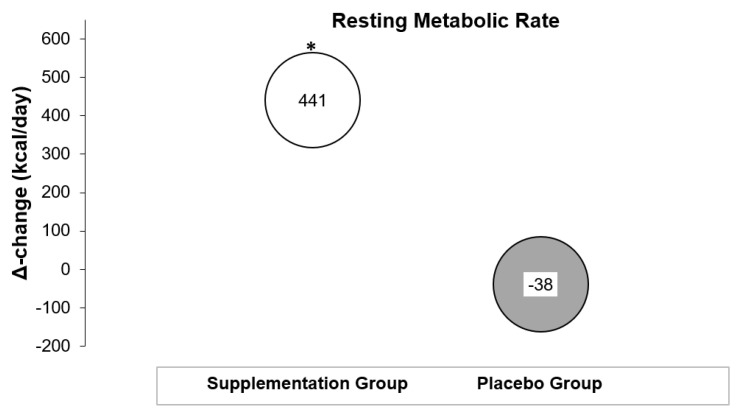
Resting metabolic rate changes after the intervention period. * Significant time × group interaction.

The results for RMR measurements are demonstrated in Table 2 and Figure 2. The analysis showed a significant interaction effect between group and time, F_1, 17_ = 4.67, *p =* 0.04. The RMR of the intervention group (M_pre_ = 1923, SE_pre_ = 129; M_post_ = 2467, SE_post_ = 141 kcal) was higher at the second assessment compared with the RMR of the placebo group, which remained unaffected (M_pre_ = 2467, SE_pre_ = 136; M_post_ = 2429, SE_post_ = 149 kcal). No main effects of group (F_1, 17_ = 3.52, *p* > 0.05) or time were found (F_1, 17_ = 3.32, *p* > 0.05).

### 3.3. Effects on Respiratory Quotient 

The results showed no significant interaction effect between group and time, F_1, 17_ = 0.63, and no main effect between groups (F_1, 17_ = 3.15, *p* > 0.05) or assessment times (F_1, 17_ = 0.40, *p* > 0.05, see Table 1).

### 3.4. Effects on Thyroid Hormones

The results for the thyroid hormones are presented in Table 2. No significant interaction effect between groups and time was found regarding FT3 F_1, 15_ = 1.11, *p* > 0.05. Furthermore, no main effect was found between groups (F_1, 15_ = 0.92, *p* > 0.05) or assessment times (F_1, 15_ = 2.71, *p* > 0.05). With regards to FT4, the analysis showed that there was no interaction effect between groups and time (F_1, 15_ = 0.06, *p* > 0.05), nor any effects of groups (F_1, 15_ = 005, *p* > 0.05). However, there was a main effect of time (F_1, 15_ = 9.64, *p* = 0.007) with participants increasing their FT4 levels by 0.760 pmol/L in the second assessment as compared with baseline. Regarding TSH, no significant interaction effect was found between groups and time (F_1, 15_ = 1.65, *p* > 0.05). Also, there were no main effects for group (F_1, 15_ = 0.21, *p* > 0.05) or assessment times (F_1, 15_ = 3.45, *p* > 0.05) 

### 3.5. Effects on Selenium and Zinc Serum Levels

Se and Zn serum results are shown in Table 2. The analysis showed a significant interaction effect between groups and time (F_1, 15_ = 11.44, *p* = 0.004) for Se. Serum Se levels of the supplementation group (M_pre_ = 83.04 μg/L, SE_pre_ = 5.71; M_post_ = 119.40 μg/L, SE_post_ = 6.23 μg/L) at the second assessment were increased compared to the serum Se levels of the placebo group (M_pre_ = 90.61 μg/L, SE_pre_ = 6.83; M_post_ = 89.58 μg/L, SE_post_ = 7.45). Moreover, a main effect of time was detected with participants in the intervention and controlled group, in total improving their serum Se levels by 17.66 μg/L in the second assessment as compared to baseline (F_1, 15_ = 10.21, *p* = 0.006). A main group effect was not observed, F_1, 15_ = 2.19, *p* > 0.05. Regarding serum Zn levels, no significant interaction effect between group and time was found (F_1, 15_ = 2.06, *p* > 0.05), nor was any effect between groups (F_1, 15_ = 0.42, *p* > 0.05) or assessment times (F_1, 15_ = 3.35, *p* > 0.05) observed.

### 3.6. Effects on Physical Fitness and Functional Capacity

The results for the physical fitness tests and functional capacity are presented in Table 3. Regarding VO_2_max, no significant interaction effect between group and time was found, F_1, 16_ = 0.15, *p* > 0.05. Moreover, no main effect between groups (F_1, 16_ = 0.15, *p* > 0.05) or assessment times (F_1, 16_ = 0.13, *p* > 0.05) was observed. With regards to the Timed Up-and-Go test, the analysis showed a significant interaction effect between group and time, F_1, 20_ = 8.15, *p =* 0.001. The post hoc tests showed that the supplementation group’s Timed Up-and-Go performance improved at the second assessment (M_pre_ = 6.85 s, SE_pre_ = 0.25; M_post_ = 6.17 s, SE_post_ = 0.22), while the placebo group’s performance decreased (M_pre_ = 6.90 s, SE_pre_ = 0.28; M_post_ = 7.06 s, SE_post_ = 0.24). However, a main effect of time (F_1, 20_ = 3.11, *p* > 0.05) and group (F_1, 20_ = 2.16, *p* > 0.05) was not observed.

Regarding the Sit-to-Stand 5 test, no significant interaction effect between group and time was found, F_1, 20_ = 0.34, *p* > 0.05. Also, no main effect between groups was observed, F_1, 20_ = 0.38, *p* > 0.05. However, a main effect was found for assessment time (F _1, 20_ = 36.38, *p* < 0.001), with the two groups demonstrating an improvement of 1.30 s at the end of the study as compared to the first assessment. 

For Sit-to-Stand 30, the analysis showed no significant interaction effect between group and time (F_1, 20_ = 0.82, *p* > 0.05) and no main effect between groups (F_1, 20_ = 0.011, *p* > 0.05). However, a main effect between assessment times was found (F_1, 20_ = 21.32, *p* < 0.001), with the two groups demonstrating an improvement of 1.78 s at the end of the study as compared to the first assessment.

No significant interaction effect between group and time was found for Sit-to-Stand 60 (F_1, 20_ = 0.91, *p* > 0.05), and no main effect between groups (F_1, 20_ = 0.28, *p* > 0.05) was observed. Yet, a main effect between assessment times was found (F_1, 20_ = 21.32, *p* < 0.001), with participants improving their performance by 3.65 s in the second assessment as compared to the beginning of the study.

For handgrip strength, no significant interaction effect between group and time was found for the left arm (F_1, 20_ = 0.25, *p* > 0.05) and no main effect between groups was observed (F_1, 20_ = 0.002, *p* > 0.05). However, a main effect between assessment times (F_1, 20 =_ 6.67, *p =* 0.018) was found, with participants improving their handgrip strength by 2.60 kg in the second assessment as compared to the beginning of the study. For the right arm, no significant interaction effect between group and time was found (F_1, 20_ = 0.00, *p* > 0.05). Also, no main effect between groups was observed (F_1, 20_ = 0.33, *p* > 0.05). A main effect between assessment times (F_1, 20_ = 8.19, *p =* 0.010) was detected, with participants improving their handgrip strength by 2.59 kg in the second assessment as compared to the beginning of the study.

## 4. Discussion

In the present study, the Zn/Se co-administration enhanced RMR of the participants and increased Se serum levels and TUG performance. However, compared to the placebo group, there were no additional benefits of Zn/Se co-administration on thyroid hormones, body composition, physical fitness, and the remaining functional capacity.

The most important finding in the present study was that Zn/Se co-administration enhanced RMR. Specifically, RMR in the supplementation group increased by 22.9%, while RMR in the placebo group decreased with weight loss. The placebo group results contrast other studies, where RMR had significantly decreased after weight loss attempts [64,65]. This difference can be attributed to the small energy deficit of the prescribed diets in the present study, combined with the study’s short intervention period [66]. In overweight premenopausal women, metabolic adaptation (i.e., a decrease in RMR at the end of the diet) has been shown to delay the time needed to achieve weight loss goals [67]. In particular, for every 10 kcal/day decrease in RMR, the time required to achieve the weight loss goal was found to be increased by one day, with the mean metabolic adaptation being approximately 50 kcal/day [67]. However, in that study measurements were conducted after a one-month stabilization phase, and it is possible that the decrease in RMR was larger immediately after the weight loss phase [67]. In another study, metabolic adaptation after weight loss in obese men and women was associated with decreased weight loss and fat-mass loss even after adjusting for confounders like dietary adherence, sex, baseline fat-mass, and randomization group [68]. This metabolic adaptation at the level of RMR was found to be persistent for as long as six years after a weight loss phase [69]. Since hypocaloric diets are the cornerstone for weight loss, the results mentioned above indicate the need for designing strategies to minimize the drop in RMR due to the metabolic adaptation observed after a diet phase. 

The average increase in RMR of the participants in the Zn/Se co-administration group was 441 kcal/day. A positive effect of Zn and Se co-supplementation on the Basal Metabolic Rate (BMR) has also been reported by Federico et al. [70] in patients undergoing chemotherapy. More specifically, after 60 days of chemotherapy, the BMR of the group that did not receive the supplements decreased by a mean of 382 calories/day at the end of the intervention, while the Zn and Se co-supplementation group maintained their baseline BMR levels. Interestingly, the increase in RMR in the present study was not accompanied by an alteration of thyroid function, indicating that other mechanisms may be responsible for the increased RMR.

In people with obesity, the increased oxidative stress, as a result of the inflammation process, can have a negative impact on mitochondrial function, with the mitochondria being unable to generate sufficient adenosine triphosphate (ATP) levels [71]. When mitochondrial dysfunction occurs, a decrease in energy expenditure can be expected since healthy mitochondria are associated with higher RMR [72]. In a recent review investigating the influence of Se on muscle function, Se supplementation was documented to increase mitochondrial biogenesis, respiratory capacity, and skeletal muscle mitochondria volume [52]. These findings are valuable since the downregulation of mitochondrial biogenesis and the decreased mitochondrial respiratory capacity and energy generation capacity are all characteristics of people with obesity due to compromised mitochondrial function [71]. Thus, with the use of Se supplementation an increase in energy consumption may be expected in people with overweight or obesity as a result of improvements in mitochondrial function [73]. Another mechanism that may be involved in the increased RMR due to Se supplementation in people with overweight or obesity involves the effects of Se on insulin resistance [57,74] and leptin [75]. 

The possible effects of Zn levels on RMR have been demonstrated in non-experimental studies. For example, Wang et al. [76] reported a positive correlation of Zn hair content with RMR. Another study found a significant association between Zn intake and RMR in overweight and obese women [10]. However, the possible mechanisms which link Zn and RMR are poorly understood. Similar to Se supplementation, Zn supplementation has also been found to positively affect insulin resistance [77] and leptin levels [78] in people with obesity. In a recent review, Zn was documented as having an essential role in the formation of a new zinc-related adiponectin, zinc-α2-glycoprotein [79]. Ζinc-α2-glycoprotein increases the binding of peroxisome proliferator-activated receptor and early b cell factor 2 to the PR domain containing 16 and uncoupling protein 1 [79]. The PR domain containing 16 and uncoupling protein 1 has previously been reported to stimulate adipose tissue browning and energy expenditure [79,80,81].

When discussing the effects of Zn and Se supplements on RMR, the two micronutrients’ role on oxidative stress management should also be addressed due to the effect of oxidative stress on mitochondrial dysfunction. Hasani et al. [82] examined the effects of Zn and Se co-supplementation in diet-induced obese rats. Thirty-two obese rats were divided into four groups and received either a high-fat diet alone or a combination of high-fat diet plus Zn, Se, or Se/Zn co-supplementation for eight weeks. At the end of the intervention, a more robust effect was detected on the antioxidant indices Glutathione peroxidase and Superoxide dismutase in the group that received Zn/Se co-supplementation compared to Zn and Se alone [82]. In addition, the co-supplementation was also more effective in decreasing the serum inflammatory markers, including Interleukin-6, malondialdehyde, and tumor necrosis factor-a [82]. According to these findings, Hasani et al. [82] concluded that the co-supplementation of Se and Zn may have a synergistic effect on inflammatory markers and oxidative stress. Notably, the baseline Se levels of the participants in our study were lower than 89 mg/L (i.e., 83.0 mg/L), which is considered as the lower limit for maximized antioxidant activity [57]. Thus, increasing Se serum levels to 90.61 mg/L at the end of the intervention may indicate an improved selenoenzyme antioxidant activity in the group that received the Zn/Se co-administration.

Some interesting findings by Hasani et al. [82] are that the rats who received the high-fat diet plus Zn significantly increased their Se serum levels and that a positive correlation was detected between Zn and Se serum levels. Furthermore, the co-supplementation of Se and Zn increased both Zn and Se serum levels [82]. In another study, the administration of Se alone in rats undergoing a Zn-deficient diet decreased the Zn deficiency, thus indicating a positive effect of Se on Zn serum levels [83]. In the present study with human participants, serum Se levels increased more in the supplementation group than in the placebo group after Se and Zn co-administration. However, Zn serum levels remained unchanged in both the intervention and placebo groups. This result aligns with the findings of a randomized controlled trial, where the combined effects of Zn and Se did not change the Zn serum status more than the placebo in overweight and obese hypothyroid patients [84].

A difference between the study conducted by Mahmoodianfard et al. [84] and the present study is that in the former, Se serum levels remained unchanged, while in the latter, they significantly increased. This difference can be explained by the different Se form used in the present study (L-selenomethionine) compared to the Se yeast used by Mahmoodianfard et al. [84]. In other studies with overweight and obese people, the effects of Zn supplementation on Zn status were found to be inconsistent, probably due to differences in the method used for the assessment of Zn (plasma and serum), baseline Zn levels, the health status of the participants, and the supplementation forms used between the studies [77,84,85,86].

The Zn/Se combination in this work did not affect thyroid hormones since both groups’ TSH, FT3, and FT4 remained unchanged. In agreement with our study, Mahmoodianfrad et al. [84] reported no significant difference between FT3, FT4, and TSH serum levels in overweight and obese hypothyroid people receiving a combination of 200 μg Se yeast/day and 30 mg of Zn gluconate/day compared to the placebo group. However, in another study, a Se supplement alone affected thyroid hormones more than the control group [57]. More specifically, Guarino et al. [57] found that 83 mcg/day of Se L-selenomethionine decreased TSH levels compared to placebo in obese people receiving a hypocaloric diet at six and twelve months. Furthermore, in a case study, supplementation of two female Zn-deficient athletes with 26.4 mg/day Zn gluconate increased T3 plasma levels in one athlete and increased T3, T4, TSH, FT3, and FT4 plasma in the other athlete at months two and four [43]. Similar to the present study, Maxwell et al. [43] reported a significant increase in the RMR. More precisely, the RMR of one participant increased by 194 kcals/day at the end of the fourth month, while in the other, it increased by 527 kcals at the end of the second month. Even though it is difficult to make any comparisons between the studies, some possible factors that could have contributed to the present study’s unchanged thyroid function are that only euthyroid individuals were included and that the Zn and Se serum levels of the participants were above deficiency cut-offs (>10.7 umol/L and >80 μg/L, respectively). In the other studies mentioned, participants with hypothyroid [84], subclinical hypothyroid, or Zn-deficient individuals were included. Moreover, any similarities between the study by Mahmoodianfrad et al. [84] and ours can be explained by the fact that the hypothyroid patients in their study were receiving l-thyroxine therapy, which could have masked the effects of supplementation on thyroid function [84]. According to a systematic review, more randomized controlled trials are needed so the combined effects of Zn and Se supplementation on thyroid function in overweight or obese people can be better clarified [33].

Even though thyroid hormones might not be the most critical hormones associated with RMR [87], any effects of Zn supplementation on thyroid hormones’ metabolism are explained through its role in the conversion of the pre-thyrotropin-releasing hormone to pro-thyrotropin-releasing hormone, T3 nuclear receptors, thyroid transcription factor 2 function, and as a cofactor of type I and II deiodinases, among others [17]. On the other hand, Se supplementation can reinforce T4 to T3 de-iodination [57] and affect the hypothalamic pituitary thyroid axis [88] and probably serum T3-binding proteins [84]. 

A lot of money is spent annually on weight loss supplements [89], and some of them may have a positive effect on weight loss. However, none of them were found to induce clinically significant weight loss [90]. Weight loss supplements’ main mechanisms of action are their effects on nutrient absorption, appetite regulation, energy expenditure modulation, fat metabolism, and carbohydrate metabolism [90]. Yet, there is a need for conducting more randomized controlled trials investigating combinations of supplements that target different weight loss mechanisms for their safety profile determination [90]. 

In the present study, the body composition in the Zn/Se co-administration group improved more than the placebo group, though this difference was not statistically significant. Body mass decreased by 1.33 kg and 0.95 kg for Zn/Se co-administration and placebo group, respectively, at the end of the intervention with a mean difference of approximately 0.4 kg between groups. This number is very close to what was reported in a meta-analysis by Abdollahi et al. [91], who reported a decrease of ~0.5 kg in healthy overweight or obese adults receiving Zn supplements compared to placebo [91]. On the other hand, when it comes to the effects of Se supplementation on body composition of overweight or obese people, mixed evidence is provided in a systematic review [33]. The authors of this review emphasized the need for more randomized controlled trials since only four studies examined the effects of Se, and only one examined the effects of a combination of Se and Zn on body composition [33].

In the present study, it is worth mentioning that the fat-free mass and muscle mass in the supplementation group increased by 0.61 kg and 0.58 kg, respectively. This was an unexpected finding, considering that the participants were on a hypocaloric diet and did not participate in a resistance training intervention. On the contrary, fat-free and muscle mass in the control group slightly decreased. Even though not statistically significant, the increased fat-free mass in the intervention group might have contributed to the effects of Se/Zn co-administration on body weight between groups. In line with the present study, positive results of the two micronutrients on fat-free mass and muscle mass have also been reported in previous studies. For example, in a cohort study, Zn was associated with muscle mass in an elderly population [92]. Moreover, in a systematic review, Zn supplementation was found to increase fat-free mass of children with growth failure [93]. In another review, Diego et al. [94] concluded that Zn can have a positive effect on muscle regeneration and biogenesis, highlighting the importance of Zn on muscle function. These effects were attributed to the role of Zn on muscle cell activation, proliferation, and differentiation [94].

Regarding Se’s effects on muscle mass, Cavedon et al. [75] found that 240 μg/day of Se as l-selenomethionine increased muscle mass significantly compared to placebo (*p* = 0.02) in overweight and obese individuals under a hypocaloric diet. In a study where the effects of Se status on skeletal muscle growth using a zebrafish model were examined, it was found that Se status can affect skeletal muscle growth by mediating protein turnover [95]. For a more detailed discussion about the effects of Zn and Se on body composition in people with overweight or obesity and some possible mechanisms underlying the effects of Zn and Se on fat loss, the readers are referred to Zavros et al. [33].

In the present study, Zn/Se co-administration was found to affect functional capacity by improving the TUG performance in the intervention group by 8.8%. Furthermore, a main effect of time was detected for all functional capacity outcomes at the end of the intervention period as compared to the baseline measurement. Notably, the participants in the supplementation group improved their performance in all tests more than the control group. However, this difference was insignificant since no time x group effects were found. One study examining the relationship between Se intake and musculoskeletal function in older adults found that low Se intake was associated with a lower TUG and handgrip performance than high Se intake [96]. Perri et al. [96] also reported a stronger association between Se intake and handgrip strength than with TUG performance. The authors assumed that this result could be attributed to the increased Se stores within the muscle and the increased complexity of the TUG test, since it has a higher cognitive demand, among others [96]. Research suggests that, similar to aging, obesity is associated with an increased risk for cognitive impairments [97]. On the other hand, Se is positively associated with cognitive function, and it is the most important nutrient within an essential trace element mixture for improvements in cognitive function [98]. Yet, since no cognitive function measurements were conducted in the present study, any relationship between Se levels, cognitive function, and TUG performance cannot be clarified, and it is proposed for future research.

A cross-sectional study examined the association between physical function (TUG performance, activities of daily leaving measurement, and handgrip and quadriceps muscle strength) and Zn levels in older people [99]. The study revealed an association between higher Zn levels and decreased functioning difficulty odds in men [99]. Interestingly, higher Zn levels were associated with increased odds of physical function difficulties in women, indicating a possible effect of sex in Zn homeostasis [99]. Due to the associations between TUG performance, quality of life [100], and medical comorbidities [62], and the positive results of Se/Zn co-administration detected in the present study, more randomized controlled trials are needed to clarify the effects of the two micronutrients in overweight and obese people’s physical function.

A limitation of the present work was that we could not include separate Se and Zn groups in order to examine the effects of each micronutrient alone. This is proposed for future research. Furthermore, although the participants were instructed not to change their physical activity levels, these were not assessed during the study. A strength of the present study was that Se and Zn serum measurements were included to determine the effects of supplementation on blood levels of these micronutrients and to compare them with other studies. Furthermore, with an individualized diet plan, daily intake of Zn and Se was strictly controlled and kept below the upper limits for the two micronutrients. An advantage of the present study was that indirect calorimetry was used for the determination of RMR instead of predictive equations, which typically either overestimate or underestimate resting energy expenditure [101].

## 5. Conclusions

The present study found a significant increase in the RMR after co-administration of 200 mcg of selenium L-selenomethionine and 25 mg of Zn gluconate, with the thyroid function hormones remaining unaffected. Furthermore, significant improvements in Se status and TUG performance were detected in the supplementation group compared to the placebo group. However, despite the larger improvements in body composition, cardiorespiratory fitness, and most of the remaining functional capacity outcomes of the intervention group, no time × group effect was found. Finally, even though the duration of the intervention, the dosages, and the forms of Se and Zn supplements used in the present study were effective, more research is needed to reveal the most effective combinations of the two micronutrients. 

## Figures and Tables

**Table 1:** Basic characteristics and body composition data divided into two groups according to the assigned intervention (bold indicates statistical significance). All data are mean ± SD.

Variables	Supplementation	Placebo	Main Effects and Interactions (*p*-Value)		
**Weight (kg)**			Time	Time × Group	Group
Baseline	83.3 ± 20.0	79.3 ± 12.2	0.098	0.776	0.593
post	82.0 ± 18.7	78.4 ± 10.9			
Δ change	−1.3 ± 3.1	−0.9 ± 2.9			
**Body mass index—BMI (kg/m^2^)**			Time	Time × Group	Group
Baseline	29.1 ± 4.4	28.3 ± 4.5	0.111	0.823	0.663
post	28.7 ± 3.9	27.9 ± 3.9			
Δ Change	−0.4 ± 1.1	−0.3 ± 1.1			
**Total Body Fat (%)**			Time	Time × Group	Group
Baseline	30.4 ± 8.0	28.3 ± 9.1	**0.014**	0.144	0.664
post	28.9 ± 7.3	27.9 ± 9.0			
Δ Change	−1.5 ± 1.7	−0.4 ± 1.7			
**Fat-free mass (%)**			Time	Time × Group	Group
Baseline	57.5 ± 14.1	56.6 ± 9.8	0.493	0.183	0.812
post	58.1 ± 14.3	56.4 ± 9.9			
Δ Change	0.6 ± 1.4	−0.2 ± 1.3			
**Muscle Mass (%)**			Time	Time × Group	Group
Baseline	54.6 ± 13.4	53.7 ± 9.4	0.498	0.186	0.810
post	55.2 ± 13.6	53.6 ± 9.5			
Δ Change	0.5 ± 1.3	−0.1 ± 1.2			

**Table 2:** Resting metabolic rate, thyroid hormones, and blood selenium and zinc levels before (baseline) and after the intervention (post) in the two groups (bold indicates statistical significance). Data are mean ± SD.

Variables	Supplementation	Placebo	Main Effects and Interactions (*p*-Value)		
**Resting Metabolic Rate (kcal/day)**			Time	Time × Group	Group
Baseline	1923 ± 440	2467 ± 367	0.086	**0.045**	0.078
post	2364 ± 410	2429 ± 484			
Δ change	441 ± 594	−38 ± 308			
**Respiratory Quotient**			Time	Time × Group	Group
Baseline	0.88 ± 0.05	0.91 ± 0.07	0.835	0.436	0.094
post	0.86 ± 0.06	0.92 ± 0.11			
Δ Change	−0.02 ± 0.07	0.01 ± 0.12			
**Free Triiodothyronine (pmol/L)**			Time	Time × Group	Group
Baseline	4.69 ± 1.02	4.17 ± 0.77	0.120	0.308	0.352
post	4.17 ± 0.65	4.06 ± 0.42			
Δ Change	−0.52 ± 0.84	−0.11 ± 0.67			
**Free Thyroxine (pmol/L)**			Time	Time × Group	Group
Baseline	15.22 ± 1.36	15.34 ± 1.82	**0.007**	0.810	0.943
post	16.04 ± 1.69	16.04 ± 2.51			
Δ Change	0.82 ± 0.86	0.70 ± 1.16			
**Thyroid-Stimulating Hormone (mIU/L)**			Time	Time × Group	Group
Baseline	1.52 ± 0.31	1.58 ± 0.34	0.083	0.218	0.650
post	1.46 ± 0.37	1.25 ± 0.456			
Δ Change	−0.06 ± 0.44	−0.33 ± 0.40			
**Selenium (** **μ** **g/L)**			Time	Time × Group	Group
Baseline	83.04 ± 13.59	90.61 ± 23.23	**0.006**	**0.004**	0.159
post	119.40 ± 23.93	89.58 ± 10.61			
Δ Change	36.36 ± 20.95	−1.03 ± 24.47			
**Zinc (umol/L)**			Time	Time × Group	Group
Baseline	11.35 ± 3.40	10.82 ± 3.45	0.087	0.171	0.525
post	11.76 ± 3.40	14.23 ± 4.65			
Δ change	0.41 ± 4.75	3.40 ± 3.26			

**Table 3:** Cardiorespiratory fitness and functional capacity for the two groups.

Variables	Supplementation	Placebo	Main Effects and Interactions (*p*-Value)		
**VO_2_max (mL/kg/min)**			Time	Time × Group	Group
Baseline	27.2 ± 7.9	26.2 ± 9.1	0.722	0.695	0.700
post	28.0 ± 5.7	26.2 ± 8.6			
Δ change	0.7 ± 5.3	−0.1 ± 2.3			
**STS-5 (s)**			Time	Time × Group	Group
Baseline	12.7 ± 2.5	13.6 ± 1.6	**0.000**	0.563	0.384
post	11.5 ± 2.4	12.2 ± 1.8			
Δ Change	−1.2 ± 0.7	−1.4 ± 1.2			
**STS-30 (rep)**			Time	Time × Group	Group
Baseline	11.7 ± 2.6	11.8 ± 0.7	**0.000**	0.376	0.735
post	13.9 ± 3.2	13.2 ± 2.2			
Δ Change	2.1 ± 1.6	1.4 ± 2.3			
**STS-60 (rep)**			Time	Time × Group	Group
Baseline	23.8 ± 5.1	23.5 ± 2.1	**0.000**	0.350	0.598
post	28.2 ± 7.2	26.4 ± 4.1			
Δ Change	4.4 ± 3.7	2.9 ± 3.6			
**Handgrip Left (kg)**			Time	Time × Group	Group
Baseline	26.4 ± 10.5	27.1 ± 10.9	**0.018**	0.616	0.966
post	29.5 ± 13.2	29.2 ± 9.3			
Δ Change	3.1 ± 5.1	2.1 ± 4.1			
**Handgrip Right (kg)**			Time	Time × Group	Group
Baseline	28.9 ± 11.4	26.1 ± 9.8	**0.010**	0.957	0.568
post	31.5 ± 14.7	28.7 ± 7.9			
Δ Change	2.5 ± 4.3	2.6 ± 4.1			
**TUG (s)**			Time	Time × Group	Group
Baseline	6.8 ± 1.1	6.9 ± 0.5	0.093	**0.010**	0.157
post	6.1 ± 0.9	7.1 ± 0.3			
Δ change	−0.6 ± 0.8 #	0.1 ± 0.4			

Abbreviations: VO_2_max, maximal oxygen uptake; STS, sit-to-stand; and TUG, timed up-and-go test. Bold means # significantly different than the placebo group. All data are mean ± SD.

## Data Availability

All data and materials of this study are available upon request.
